# Time-Course Expression Profiles of Hair Cycle-Associated Genes in Male Mini Rats after Depilation of Telogen-Phase Hairs

**DOI:** 10.3390/ijms10051967

**Published:** 2009-04-28

**Authors:** Aya Umeda-Ikawa, Isao Shimokawa, Kunio Doi

**Affiliations:** 1 Department of Veterinary Pathology, Graduate School of Agricultural and Life Sciences, the University of Tokyo, 1-1-1 Yayoi, Bunkyo, Tokyo 113-8657, Japan; E-Mail: umeda_aya@yahoo.co.jp; 2 Department of Respiratory and Digestive Medicine, Division of Experimental Medicine, Pathology, and Gerontology, Nagasaki University School of Medicine, 1-12-4 Sakamoto, Nagasaki, Nagasaki 852-8523, Japan; E-Mail: Shimo@nagasaki-u.ac.jp; 3 Nippon Institute for Biological Science, 9-2221-1, Shin-Machi, Ome, Tokyo 198-0024, Japan

**Keywords:** GH-deficient Mini rats, dorsal skin, hair cycle, microarray analysis

## Abstract

Jcl:WistarTGN(ARGHGEN)1Nts rat (Mini rat) is a growth hormone (GH)-deficient transgenic rat. The hair cycle in the dorsal skin of male Mini rats enters a long-lasting telogen phase after eights weeks of age, but depilation can induce a transient hair cycle again. In this study, a time-course profiling of genes expression was done on the dorsal skin of male Mini rats along the progression of depilation-induced hair cycle using DNA microarray analysis. As a result, 1,215 probe sets including 1,171 hair cycle-related ones showed more than 3-fold changes in expression compared with that in before-depilation telogen phase. The present data will contribute to elucidating the mechanisms of hair cycle regulation and should lead to the identification of novel molecular targets for hair growth and/or depilation agents.

## 1. Introduction

The Jcl:WistarTGN(ARGHGEN)1Nts rat (Mini rat) is a transgenic rat in which the growth hormone (GH) expression is suppressed by the presence of an antisense transgene for the rat GH gene located under the GH promoter gene expressed in the pituitary [1]. Compared to male Wistar rats, the parental strain of Mini rats, male Mini rats show reduced plasma levels of GH (approximately 50 % of Wistar rats) [2] and insulin-like growth factor (IGF)-1 (approximately 50 % of Wistar rats) [3], and their body mass is half that of Wistar rats. Mini rats have thinner skin with less collagen, more abundant subcutaneous adipose tissue, and small-sized sebaceous glands compared, though there are no essential differences in the skin structures between them and Wistar rats [3].

In rodents, hair follicle morphogenesis starts late in embryogenesis, and postnatal hair follicles undergo a cyclic process of active growth (anagen), follicle regression (catagen), and resting (telogen), followed by the next anagen [4], and this cyclical growth of hair follicles persists throughout their life. The hair cycle pattern of Mini rats is very different from that of Wistar rats. Namely, two cycles of four weeks each are observed in both strains during the first eight weeks after birth, but the cycle enters a long-lasting telogen phase until at least 24 weeks of age in Mini rats, while the 3rd cycle starts in Wistar rats immediately afterwards, though it is unsynchronized [3]. The dorsal skin hair follicles of male Mini rats under such long-lasting telogen phase are anagen-inducible, and a transient cycle is able to be induced again by hair plucking [5] or by traumatic stimulus [6]. This suggests that Mini rats may lack an adequate internal stimuli or signals essential to transition from telogen to anagen after eight weeks of age, but certain external stimuli are able to work as a trigger driving hair growth cycle.

It has been stressed that time-course profiling of the whole skin, which involves the epithelial and mesenchymal components, is needed for comprehensive characterization of the complex molecular changes in hair growth and cycling, and DNA microarray analysis is one of the most powerful approaches which can detect changes in the expression of large numbers of genes. Recently, such profiling has been carried out on mice in order to understand the molecular mechanisms governing hair growth and cycling [7–9]. However, to date, no systematic analysis of gene expression in the hair follicle and during the growth cycle has been done in rats.

In the present study, DNA microarray analysis was carried out on the dorsal skin of male Mini rats in order to find a clue to clarify the time-course gene expression profile along hair growth following depilation of telogen-phase hairs at 10 weeks of age, by comparing the after-depilation groups (five time points) with the before-depilation control group.

## 2. Results and Discussion

### 2.1. Changes in histology and depth (length) of hair follicles

**Figure 1.**
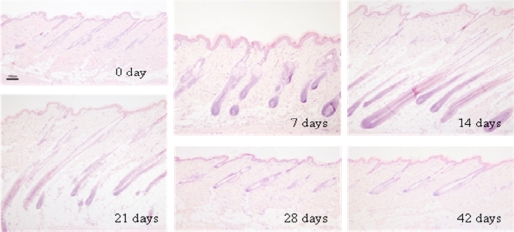
Changes in histology of dorsal skin of male Mini rats after depilation of telogen-phase hairs at 10 weeks old. HE stain, Bar = 100 μm.

**Figure 2.**
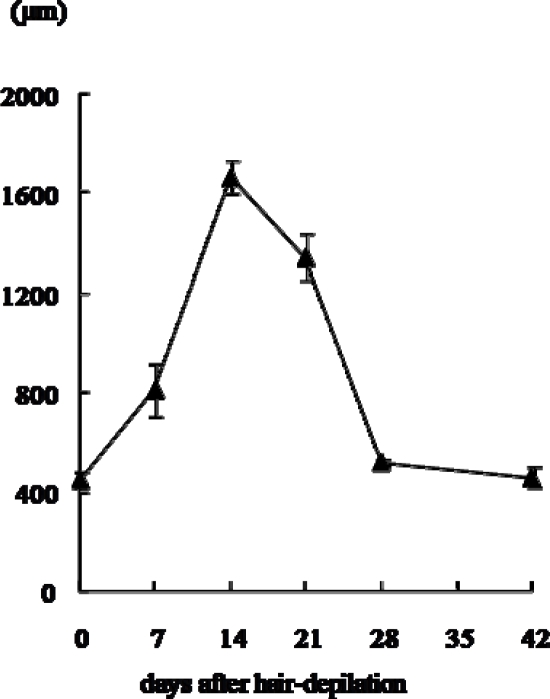
Changes in depth (length) of hair follicles in dorsal skin of male Mini rats after depilation of telogen-phase hairs at 10 weeks of age. The data represents mean + standard deviation of three animals except for the group at 42 days after depilation (two animals).

Changes in histology and depth (length) of hair follicles are shown in Figures 1 and 2. The hair growth showed basically neither individual nor regional differences in both macroscopic and microscopic observations, suggesting that the dorsal skin hair of Mini rats lacks the wave pattern usually seen in normal rats [10].

The hair follicles were in telogen phase, in the before-depilation control group (10 weeks old) as reported by Ikawa *et al.* [3]. The regrowth of hair follicles with elongation of hair shafts was detected after the hair plucking, as previously reported by Umeda-Ikawa *et al.* [5]. Namely, at seven days after hair plucking, the length of hair follicles became longer than that in the control group. Enlarged dermal papilla of loose consistency were noted, and the bulbs and dermal papilla of many hair follicles resided in the border between the dermis and subcutis. At 14 days, hair follicles were fully developed and hair shafts were more elongated compared with those at seven days. Many bulbs resided deep in the subcutis above the panniculus carnosus, and both the hair shaft and inner root sheath reached a level around the insertion of the sebaceous gland in many follicles. At 21 days, the length of hair follicles became shorter than that at 14 days. The follicles had smaller and narrower bulb which remained in the subcutis. At 28 and 42 days, short hair follicles were fully surrounded by interfollicular dermal fibroblasts, and the length of hair follicles was almost the same as that of the before-depilation control group. Taking these findings and previous reports [3,4] together, the hair cycle was considered to be early anagen, anagen, catagen, telogen and telogen at 7, 14, 21, 28 and 42 days after the hair plucking, respectively.

### 2.2. Gene expression profiles

The data of DNA microarray analysis on the dorsal skin of male Mini rats between the before-depilation control group (10 weeks old: telogen phase) and the after-depilation groups (7 days: early anagen phase; 14 days: anagen phase; 21 days catagen phase; 28 and 42 days: telogen phase) was compared. As a result, 1,215 probe sets including 1,171 hair cycle-related ones showed increases or decreases of more than 3-fold after the hair plucking, and some of them are categorized according to their function (Table 1).

**Figure 3.**
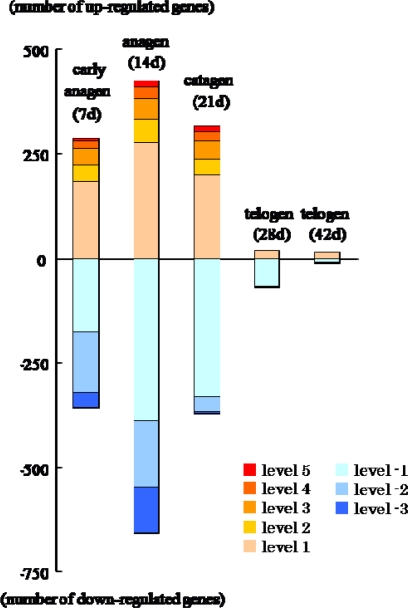
Expression pattern of genes according to their fold-change levels at each phase of hair cycle in dorsal skin of male Mini rats after depilation of telogen-phase hairs at 10 weeks old.

**Figure 4.**
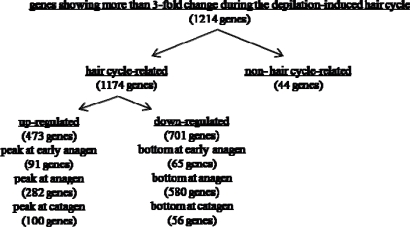
The number of genes showing the highest or lowest expression at each phase of hair cycle in dorsal skin of male Mini rats after depilation of telogen-phase hairs at 10 weeks old.

Compared with the before-depilation telogen phase, prominent changes were noted during the depilation-induced hair cycle, especially in the anagen phase, whereas there were only a small differences observed between the before- and after-depilation telogen phases (Figure 3). Namely, 859 out of 1,171 hair cycle-associated genes reached their highest or lowest expression levels at anagen phase (Figure 4), and 214 of them showed a more than 10-fold increase (levels 3–5) or decrease (level-3) (Figure 3).

**Figure 5.**
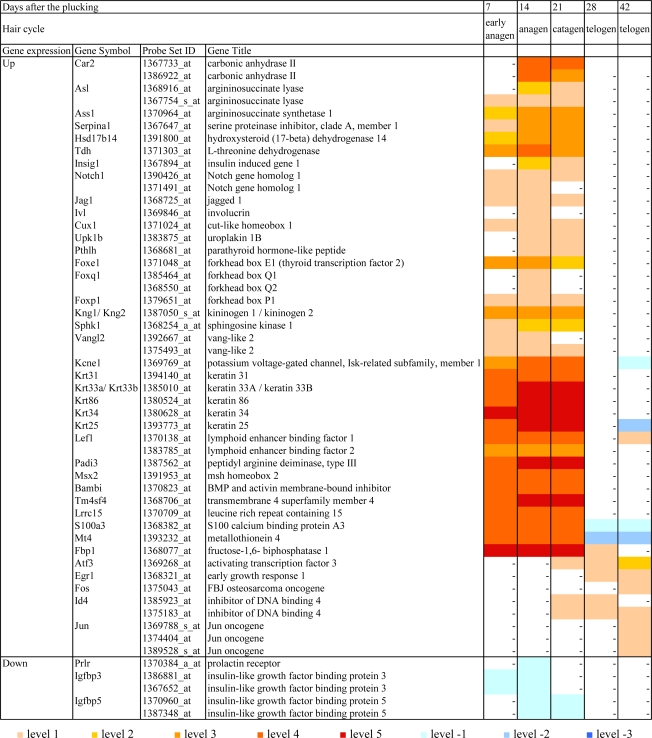
Expression pattern of main genes in dorsal skin of male Mini rats after depilation of telogen-phase hairs at 10 weeks old.

Figure 5 includes the main genes which showed up- or down-regulation during the depilation-induced hair cycle. Many genes regulating cell cycle, cell division and/or mitosis reached their peak expression at early anagen phage and those regulating transcription or cell adhesion from early anagen to anagen phases, respectively. The expression of genes controlling amino acid metabolism and signal transduction including G-protein coupled receptor signaling pathway reached the highest level at anagen phage, and that of genes involved in epidermal development, cell communication or proteolysis was strongly up-regulated from anagen to catagen phases. In addition, as shown in Figure 5, the expression of such genes as Car2 and Asl was highly up-regulated from anagen to catagen phases, but it showed no change in early anagen phase. Such expression profiles of Car2 and Asl are thought to be a response to steroid hormone stimulus, and up-regulated Car2 and As1 are considered to be related to the induction of hair follicle apoptosis. Some genes related to the Wnt signaling pathway, which is well known to drive the hair cycle [11,12], as well as those related to transforming growth factor (TGF)-β families, were up-regulated. Most of them peaked at anagen phase. Notch gene families are suggested to be one conceivable molecular pathway for the control of epithelial cell lineages of hair follicles [11]. Notch1 and its ligand Jag1 were mildly up-regulated from early anagen to catagen phases. In addition, some genes such as Ivl, Cux1, and forkhead boxes, which are known to be related to hair follicle differentiation and morphogenesis, were also up-regulated, and Kcnel which is involved in epithelial cell maturation also showed high expression from early anagen to catagen phases.

As shown in Figure 5, the following genes were markedly up-regulated (level 4 or 5) through the depilation-induced hair cycle, and returned to the levels of the before-depilation telogen phase again; some types of keratins (Krt31, Krt33a, Krt86, Krt34 and Krt25), Lef1, which is implicated in hair shaft differentiation, Wnt-β-catenin-Lef1, which is a well-defined key pathway in hair cycle control, Padi3, of which expression in epidermis and hair follicles may be involved in the hair follicle program of differentiation, Msx2, which contributes to anagen maintenance, Bambi, which is involved in TGF-β receptor signaling pathway, Tm4sf4, of which expression may control the cell proliferation, Lrrc15, which may have function in cell communication, S100a3, which plays an important role in calcium-dependent processes leading to hair shaft formation, Mt4, which may act in cellular metal ion homeostasis, and Fbp1, which is involved in gluconeogenesis (Netaffx) [12–14].

Moreover, although infrequent, some genes such as Atf3, Egr1, Fos, Id4 and Jun, which are listed in Table 1, were mildly up-regulated in the catagen phase and/or telogen phase after the hair plucking. These changes are thought to be age-related.

On the other hand, as shown in Table 1, genes which indicate negative cell growth tended to be down-regulated during the depilation-induced hair cycle. Genes involved in immune response were also suppressed during the depilation-induced hair cycle compared with the before-depilation telogen phase, and this may reflect anagen-associated immunosuppressive activities [11]. The expression of most down-regulated genes reached the lowest level at anagen phase, but that of genes related to steroid biosynthetic or metabolic process, including Soat1 and prolactin receptor, reached the lowest level somewhat later, i.e. at anagen and/or catagen phases. IGF-1 has pleiotropic effects over cell proliferation, tissue remodeling, and hair cycle control as well as differentiation within the hair-producing follicles [15]. In the present study, although there were little changes in the expression of IGF-1 and its receptors in the dorsal skin, the expression of such binding proteins as Igfbp3 and Igfbp5 which bind to Igf and and regulate IGF’s action was decreased in early anagen and anagen phases (Igfbp3) or in catagen and telogen phases (Igfbp5) (Figure 5). However, the effect of reduced plasma levels of GH and IGF-1 in Mini rats on the expression profiles of hair cycle-associated genes remains unclear.

The skin is a complex tissue in which multiple biological processes occur simultaneously, potentially masking each other at the level of gene expression. Nonetheless, as mentioned above, the results obtained successfully present a simple outline of gene expression profiles along the hair cycle progression after the depilation of telogen-phase hairs, probably because of their high magnitude of changes. In addition, in the present study, Gsg1 (germ cell associated 1) was not expressed in telogen phase but was markedly up-regulated during the depilation-induced hair cycle. Such change in Gsg1 expression has not previously been reported, and this may provide a useful seed for further evaluation of the event occurring during the hair cycling.

Depilation of telogen-phase hairs has been studied in mouse models to synchronize the development of anagen hair follicles. This technique has been used with infant C57BL/6 mice for their advantage in detecting telogen phase by pink skin color [4,11]. However, it is difficult in matured rodents, because the first two hair-growth cycles occur in synchronized waves, then unsynchronized after the second cycle [4,7]. However, the hair follicles of male Mini rats are uniformly under anagen-inducible telogen phase after 8 weeks old. Therefore, adult male Mini rats are useful as a depilation-induced hair cycling model because we are able to eliminate non cyclical changes peculiar to growth alteration of the skin usually seen at puberty in mice and rats. Thus, the characteristic hair cycle in male Mini rats will offer a powerful tool for investigating the molecular nature and organization of the so-called “hair cycle clock” [12], or in search for anagen-inducible drugs.

## 3. Materials and Methods

### 3.1. Animals

Seventeen 10-week-old male Mini rats (Jcl:WistarTGN(ARGHGEN)1Nts rats) were used. The animals were kept using isolator caging systems (Niki Shoji Co., Tokyo, Japan) in an animal room controlled at 24 ± 1 °C with a relative humidity of 55 ± 5 % and 12 h/12 h light/dark cycle. They were fed commercial pellets (CRF-1, Oriental Yeast Co., Tokyo, Japan) and tap water *ad libitum*.

### 3.2. Treatments

The protocol of this study was approved by the Animal Use and Care Committee of the Graduate School of Agricultural and Life Sciences, the University of Tokyo.

Dorsal skin of 14 10-week-old male Mini rats, in which hair follicles were under telogen phase [3], were artificially plucked using depilation sheets (ANSCHONE Lepias Co., Ltd., Osaka, Japan) under ether anesthesia. Then, rats were killed by exanguination under ether anesthesia at 7, 14, 21 and 28 days (three animals each) and 42 days (two animals) after the hair plucking, respectively. The remaining three 10-week-old animals were killed in the same way before the hair plucking and used as controls. At necropsy, after shaving dorsal pelage, the dorsal skin (about 1 cm × 1 cm) was bisected with the vertebral line and collected for histological examinations and total RNA isolation.

Half of the skin sample collected from each animal was cut into strips parallel to the longitudinal axis of the body and fixed in 10 % neutral-buffered formalin or histological processing. The other half was diced finely and soaked in RNAlater (Ambion Inc., Austin, TX, USA) to prevent RNA degeneration, and total RNA was isolated from the skin samples using RNeasy Fibrous Tissue Mini Kit (Qiagen, Tokyo, Japan) according to the manufacturer’s instructions.

### 3.3. Histology

Paraffin sections (4 μm) of the dorsal skin were stained with hematoxylin and eosin (HE) for histological examinations. The depth (length) of hair follicles was measured on HE- stained sections using a micrometer under a light microscope as previously reported [3]. Ten randomly chosen hair follicles/2 sections/animal were measured, and the mean value was calculated for each animal. Hair cycle was judged based on the morphology and the depth (length) of hair follicles as previously reported [3,4].

### 3.4. Microarray analysis

DNA microarray analysis was conducted on the above-mentioned dorsal skin of male Mini rats using GeneChip Rat Genome 230 2.0 Arrays (Affymetrix, Santa Clara, CA, USA) containing 31,099 probe sets to determine the gene expression profiles. A chip was employed for each total RNA sample derived from a rat. The procedure was conducted according to the manufacturer’s instruction using Superscript Choice System (Invitrogen, Carlsbad, CA, USA) and T7-(dT)24-oligonucleotide primer (Affymetrix) for cDNA synthesis of biotin-labeled c RNA. The fragmented cRNA was hybridized to the array for 18 hr at 45 °C at 60 rpm, after which the array was washed, stained with streptavidin-phycoerythrin, and then scanned with the GeneArray scanner 3,000 (Affymetrix).

### 3.5. Microarray data analysis

Analyses of the resulting images and data files were performed using Affymetrix Microarray Suite 5.0 (Affymetrix) and NetAffx website (http://www.affymetrix.com/analysis/index.affx). Before comparing any measurements, all the microarray data were scaled by global normalization where the mean signal intensity of earch data was adjusted to 100. We extracted the probes with present call for all replicates in at least one of the groups, and excluded the probes in which the group average intensities were lower than the target value (= 100) in all groups. When more than half of the detection flags were not present in both the before-depilation and the after-depilation groups, the group was judged to have no change. The fold change was calculated using the group average intensity, and more than 3-fold increase or decrease in the expression compared with the before-depilation group was used as criteria for a meaningful change. To simplify the data analysis, the degree of changes was classified as follows; level 5: more than 100-fold increase, level 4: more than 30-fold increase, level 3: more than 10-fold increase, level 2: more than 5-fold increase, level 1: more than 3-fold increase, level 0: no change, level-1: less than 3-fold decrease, level-2: less than 6-fold decrease, and level-3: less than 10-fold decrease.

## 4. Conclusions

The hair follicles of GH-deficient male Mini rats remain in a long-lasting telogen phase after 8 weeks of age, but depilation of telogen-phase hairs can induce a transient hair cycle again. Utilizing such characteristics of male Mini rats, the present study clarified a time-course expression profile of hair cycle-associated genes in the dorsal skin after the depilation of hairs under a long-lasing telogen phase. To our knowledge, this is the first report of DNA microarray analysis done on hair cycle-associated genes in the adult rat skin. Male Mini rats will offer a powerful tool for investigating the molecular nature and organization during the hair cycling, and for identification of novel molecular targets for hair growth and/or depilation drugs. Further studies on the special expression patterns of the above-mentioned hair growth-associated genes should be done in near future.

## Figures and Tables

**Table 1.** Selected genes categorized according to their function that showed more than 3-fold changes in dorsal skin of male Mini rats after depilation of telogen-phase hairs at 10 weeks old.

**Category**	**Gene symbol**
**Genes up-regulated during the depilation-induced hair cycle**	
Cell cycle/ tissue regeneration	3 cyclins, 4 cell division cycles, 3 tubulins, 3 kinesin families, Tpd52l1, Pttg1, Nuf2, Tm4sf4, Tm4sf1
Proliferation/differentiation/development	Cml5, Hspa2, Kng1, Sphk1, Mycn, Pthlh, Upk1b, Cux1, Tyrp1, Vangl2, Dlx3, Stmn1, Marcksl1, Pdlim7
Keratin/ keratinocyte differentiation	6 keratins, Sprr1al, Ivl, Notch1
Translation	5 eukaryotic translation initiation factors, Cars, Yars, Rpl7
Regulation of transcription	Foxe1, Foxq1, Foxp1, Sox4, Csdc2, Top2a, Myo5a, Mafb, Trps1, Cited4, Dnmt1, E2f5, Fhl2, Hmgb2, Pcgf6
Wnt/TGFβ signaling pathway	Lef1, Bambi, Msx1, Msx2, Bmp4, Csnk1e, Gpc3, Fzd1, Stra6, Mitf, Smad7, Sostdc1, Wnt4, Tcf7
Transport	11 solute carrier families, Abcg2, Fxyd4, Kcne1, Nup210, Selenbp1, Scg5, Ucp2, 2 ATPases, Bspry
Signal transduction	Itsn1, Gprc5a, Gpsm2, Ednrb, P2ry5, Ptger4, Gpr56, Nradd, Farp1, Mfhas1
Amino acid metabolic process	Ggt1, Acy3, Gnmt, Cbs, Gss, Gclc, Cth, Otub2
Carbohydrate metabolic process	Fbp1, 2 protein phosphatases, Hexb
Steroid hormone related	Hsd17b14, Tdh, Serpina1, Car2, Ass1, Asl, Arg1, Insig1, Adipor2, Fads3
Cell Adhesion	3 collagens, Dsc2, Gpnmb, Spon1
Proteolysis	3 cathepsins, Cpm, Capn8, Lap3, Pitrm1, Metap2, Prep, St14, Atg4b
Cell communication	Lrrc15, Gjb2, Gjb6, Jag1
Protein modification process	Padi1, Padi3, Padi4
Calcium ion binding	S100a3, S100a7a, Tesc, Tchh, Mt4
**Genes down-regulated during the depilation-induced hair cycle**	
Cell cycle arrest	Ak1, Cgref1, Pmp22, Nbl1, Dst
Anti-apoptosis/regulation of apoptosis	Eef1a2, Sod2, Cryab, Tsc22d3, Vnn1, Cabc1, Cidea, Nol3, Cck
Negative regulation of cell growth	4 insulin-like growth factor binding proteins, Dab2, Gas6
Immune response	5 chemokine ligands, 4 CD antigens, 4 Fc receptors, 4 RT1-class II, 7 complements, Il1b, Hla-dma
Regulation of transcription	Abra, Ldb3, Six1, Satb1, Cebpd, Dbp, Synpo2, Rxrg, Tcea3, Myf6, Tbx15, Lass5, Aebp1, Rora, Lrrfip1, Tnxa
Wnt/TGFβ signaling pathway	Cav1, Cav2, Cpz, Grem1, Grem2, Sfrp4
Transport	4 ATPases, 2 calcium channels, 3 sodium channels, 6 solute carrier families, 4 transmembrane proteins, Mup5, Fxyd1, Cp, Serpinh1, Ap1s2, Rbp7, Clic2
Signal transduction	Hspb6, Agtr1a, Mfap4, Lpar1, Rgs10, Cxcr7, Plcb4, Obscn
Carbohydrate metabolic process	5 protein phosphatases, 2 pyruvate dehydrogenase kinases, Pgm1, Phka1, Pygm, Gpd1, Gfpt2, Agl
Fatty acid biosynthetic/metabolic process	Ankrd23, Cpt1b, Fabp3, Acadm, Crot, Ptgs1, Phyh
Steroid biosynthetic/metabolic process	Ebp, Soat1, Hmgcr, Prlr, Prkaa2, Sult1a1, Vldlr
Cell adhesion	6 collagens, Lox, Emb, Ddr2, Itgbl1, Epdr1, Hrc
Proteolysis	Mmp2, Ctsk, Cpa3, Mcpt1, Pcolce, Ctss, Dpp7, Pgcp
Muscle contraction	9 myosin polypeptides, 6 troponins, Mybpc2, Mylk2, Myom1, Tmod1, Tpm1
**Genes up-regulated with aging**	
Regulation of transcription	Jun, Fos, Atf3, Id4, Egr1
